# Early Fault Diagnosis of Bearings Using an Improved Spectral Kurtosis by Maximum Correlated Kurtosis Deconvolution

**DOI:** 10.3390/s151129363

**Published:** 2015-11-20

**Authors:** Feng Jia, Yaguo Lei, Hongkai Shan, Jing Lin

**Affiliations:** State Key Laboratory for Manufacturing Systems Engineering, Xi’an Jiaotong University, No. 28 Xianning West Road, Xi’an 710049, China; E-Mails: jiafeng1237@sina.com (F.J.); hongkai1119@163.com (H.S.); jinglin@mail.xjtu.edu.cn (J.L.)

**Keywords:** maximum correlated kurtosis deconvolution, spectral kurtosis, rolling element bearing, early fault diagnosis

## Abstract

The early fault characteristics of rolling element bearings carried by vibration signals are quite weak because the signals are generally masked by heavy background noise. To extract the weak fault characteristics of bearings from the signals, an improved spectral kurtosis (SK) method is proposed based on maximum correlated kurtosis deconvolution (MCKD). The proposed method combines the ability of MCKD in indicating the periodic fault transients and the ability of SK in locating these transients in the frequency domain. A simulation signal overwhelmed by heavy noise is used to demonstrate the effectiveness of the proposed method. The results show that MCKD is beneficial to clarify the periodic impulse components of the bearing signals, and the method is able to detect the resonant frequency band of the signal and extract its fault characteristic frequency. Through analyzing actual vibration signals collected from wind turbines and hot strip rolling mills, we confirm that by using the proposed method, it is possible to extract fault characteristics and diagnose early faults of rolling element bearings. Based on the comparisons with the SK method, it is verified that the proposed method is more suitable to diagnose early faults of rolling element bearings.

## 1. Introduction

As rolling element bearings are widely used in rotating machinery and one of the most easily damaged components as well, their early fault diagnosis has attracted lots of attention [1,2,3,4]. Typically, the early faults of bearings are difficult to detect by extracting fault characteristics from the vibration signals with low signal-to-noise ratios [5]. Therefore, how to effectively extract weak fault characteristics is the key step for further identifying the early faults of bearings. In order to solve this problem, researchers have investigated plenty of signal processing techniques [6,7,8,9,10]. Among these techniques, envelope analysis is widely used because of its ability to isolate the impulse responses of bearings. It determines the locations and types of the bearing faults by extracting the fault information from the interested frequency band of the signals and reduces the influence of non-fault periodic components [11]. However, the selection of the suitable analysis frequency bands always depends on diagnostic expertise, which can greatly influence the accuracy of the analysis results. Therefore, spectral kurtosis (SK) was developed to identify the frequency bands with a great quantity of impulses [12].

Antoni [13] further studied the theory of spectral kurtosis and proved that SK could detect the frequency bands with the impulsiveness excited by defects of the components, and then applied the spectral kurtosis in mechanical fault diagnosis. However, the drawback of SK is that the method may fail in effectively detecting transients with a low signal-to-noise ratio. To remedy this drawback, Wang *et al.* [14] proposed an adaptive spectral kurtosis method for bearing fault diagnosis and the method could determine the optimal bandwidth and center frequency adaptively. Wang *et al.* [15] developed an enhanced SK method which calculates kurtosis values based on the power spectrum and wavelet packet nodes at different depths. Xu *et al.* [16] applied the periodic component to aperiodic component ratio for finding the frequency band with periodic impulses of SK and the results showed the effectiveness of the method. Wang *et al.* [17] combined minimum entropy deconvolution (MED) and SK for extracting weak fault characteristics of the bearings, and the results showed that the method performed better than the wavelet transform and ensemble empirical mode decomposition. Fan *et al.* [18] developed a method to enhance the capability of SK by using cepstrum pre-whitening and MED, so that the impulses of the original signals can be effectively deconvolved from the effect of the transmission path. Through the literature review, we notice that MED enhances the performance of SK because it could sharpen the impulses and increase the kurtosis of the signals. However, MED may fail to extract the desired ones, especially in the diagnosis of low speed bearings, since MED ignores the periodic nature of bearing signals [19]. 

To take advantage of the periodic nature of the bearing fault signals and diagnose the early faults accurately, this study proposes an improved SK method by using the maximum correlation kurtosis deconvolution (MCKD) technique. First, MCKD, as an improved MED method, is used to highlight the periodic impulse components of the vibration signals. Then SK is applied to select the resonant frequency band of the signal filtered by MCKD and generate the envelop spectrum for diagnosing the faults. Harnessing the advantages of MCKD and SK, the proposed method is expected to effectively extract the weak fault characteristics and identify the early faults of the bearings. The remainder of the paper is organized as follows: Section 2 briefly introduces MCKD and SK. Section 3 describes the proposed method. In Section 4, a simulation is used to illustrate the ability of the proposed method in detecting the resonant frequency band and extracting the fault characteristics from the signal overwhelmed by heavy noise. In Section 5, the proposed method is applied to diagnose the early bearing faults of wind turbines and rolling mills, respectively. Through the results of the two diagnosis cases, the effectiveness of the proposed method is verified. Conclusions are drawn in Section 6.

## 2. Theoretical Background

### 2.1. MCKD Technique

MCKD, proposed by McDonald *et al.* [19], takes advantage of the periodic nature of bearing faults as well as the impulse-like vibration behavior associated with these faults. It aims at selecting a finite impulse response filter (FIR) to maximize the correlated kurtosis of signals and encourage its periodicity. 

When a fault occurs, we can define discrete signal *x*(*n*) as the response of the bearing excited by the fault impulse signal *y*(*n*). MCKD searches for a FIR filter *w*(*l*) to maximize the correlation kurtosis of the signal *y*(*n*) recovered from the input signal:

(1) $$y(n)=\sum_{k=1}^{L}w(k)x(n-k+1)$$

where *l* = 1, 2, Λ, *L*, and *L* is the length of the FIR filter.

The correlation kurtosis is defined as:

(2) $$CK_{M}(T)=\frac{\sum_{n=1}^{N}(\prod_{m=0}^{M}y(n-mT){)}^{2}}{{\left(\sum_{n=1}^{N}y^{2}(n)\right)}^{M+1}}$$

where *T* is the period of the impulses and *M* is the shift number.

The optimization function of MCKD is expressed as:

(3) $$MCKD_{M}(T)=\underset{w(l)}{\max}\frac{\sum_{n=1}^{N}(\prod_{m=0}^{M}y_{n-mT}{)}^{2}}{{\left(\sum_{n=1}^{N}y_{n}^{2}\right)}^{M+1}}$$

The optimization function is to find the optimal filter which maximizes the correlation kurtosis *CK_M_*(*T*), namely:

(4) $$\frac{d}{dw(l)}CK_{M}(T)=0$$

The results of Equation (4) are the coefficients of the *w*(*l*) which can be expressed by a matrix form as follows:

(5) $$w=\frac{{\left\|y\right\|}^{2}}{2{\left\|B\right\|}^{2}}{\left(X_{0}X_{0}^{T}\right)}^{-1}\sum_{m=0}^{M}X_{mT}A_{m}$$

where $X_{mT}=\left[\begin{array}{cccc} x_{1-mT} & x_{2-mT} & \cdots & x_{N-mT}\\ 0 & x_{1-mT} & \cdots & x_{N-1-mT}\\ \vdots & \vdots & \ddots & \vdots \\ 0 & 0 & \cdots & x_{N-L-mT+1} \end{array}\right]\begin{array}{c} L\times N \end{array}$, $A_{m}=\left[\begin{array}{c} y_{1-mT}^{-1}(y_{1}^{2}y_{1-T}^{2}\cdots y_{1-mT}^{2})\\ \vdots \\ y_{N-mT}^{-1}(y_{N}^{2}y_{N-T}^{2}\cdots y_{N-mT}^{2}) \end{array}\right]\begin{array}{c} N\times 1 \end{array}$ and $B=\left[\begin{array}{c} y_{1}y_{1-T}\cdots y_{1-MT}\\ \vdots \\ y_{N}y_{N-T}\cdots y_{N-MT} \end{array}\right]\begin{array}{c} N\times 1 \end{array}$.

### 2.2. Spectral Kurtosis

The spectrum kurtosis method was first introduced by Dwyer to overcome the problems of the power spectral density method in indicating the transients of the signals in the applications [12]. Antoni further studied on the theory of SK and gave the formal definition by theoretical analysis.

The signal *Y*(*t*) is defined as the response of a system by a signal *X*(*t*), which is expressed as:

(6) $$Y(t)=\int_{-\infty }^{+\infty }\begin{array}{c} e^{2\text{π}ft}H(t,f)dX(f) \end{array}$$

where *H*(*t*, *f*) is the time-varying transfer function. Since *e*^2π*ft*^*H*(*t*, *f*)*dX*(*f*) is the result of a narrow-band filter centered on frequency *f*, *H*(*t*, *f*) can be regarded as the complex envelope of the signal *Y*(*t*) at *f*.

The fourth-order spectral cumulant of *Y*(*t*) is defined as:

(7) $$C_{4Y}(f)=S_{4Y}-2S_{2Y}^{2}(f)$$

where *S*_2*Y*_(*f*) is calculated by *S*_2*nY*_(*t*, *f*), and *S*_2*nY*_(*t*, *f*) is the 2*n* order transient moment.

Then SK is defined as the normalized cumulant calculated by:

(8) $$K_{Y}(f)=\frac{C_{4Y}(f)}{S_{2Y}^{2}(f)}=\frac{S_{4Y}(f)}{S_{2Y}^{2}(f)}-2$$

SK is affected by the chosen window length. In order to determine the window length of SK, Antoni used the short-time Fourier transform to compute various window lengths of SK and selected the interested frequency band where the kurtosis was maximized. This technique led to the concept of the kurtogram, which is a diagram presenting values of SK calculated by a series of filters with different parameters of center frequency and bandwidth. Then the fast kurtogram was developed by Antoni to promote the calculation efficiency [13]. It is used to process the signals in this study.

## 3. The Proposed Method

Spectral kurtosis has been applied to rotating machinery fault diagnosis for years and has achieved good results [14]. In the early stage of bearing faults, however, using SK to detect the impulses of the signals is difficult since potential periodic impulses are often overwhelmed by unexpected heavy noise [15]. To enhance the effect of SK, some preprocessing methods are needed to improve the signal-to-noise ratio and highlight the fault impulses, which would offer a great help for diagnosing the early faults of rolling element bearings. MED has shown its effectiveness in deconvolving the impulses from a mixture of response signals and has been used to enhance the performance of SK in bearing fault detection and diagnosis. However, if the fault impulses are submerged in noise, MED tends to only deconvolve a single impulse or a selection of impulses instead of the desired periodic impulses. To solve this problem, MCKD is developed by considering the periodic nature of the fault impulses and its superiority to MED has been illustrated [19]. Therefore, MCKD may greatly improve the performance of SK and accurately diagnose the early faults of the bearings.

**Figure 1:**
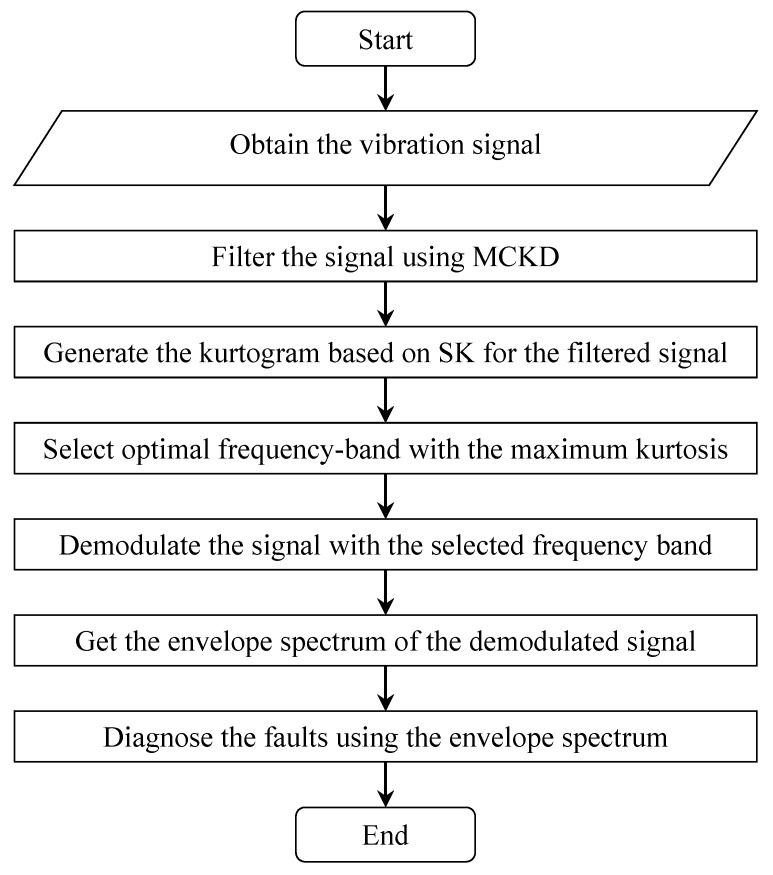
Flow chart of the proposed method.

Based on the analysis above, this study proposes a new method for early fault diagnosis of rolling element bearings. The method harnesses the ability of MCKD in highlighting the periodic fault transients and the ability of SK in locating these transients from the frequency domain. The flow chart of the proposed method is presented in Figure 1 and the details are described as follows.

(1) Obtain an original vibration signal *x*(*n*) of the bearings and filter it by MCKD. Since periodic impulses excited by an incipient defect of bearings are weak and always masked by other components of the vibration signal, MCKD is used to filter the signal *x*(*n*) and enhance these periodic fault impulses. The filter coefficient *w*(*l*) is determined by Equation (5) and the filtered signal *y*(*n*) is the convolution result of *x*(*n*) with *w*(*l*).
(2) Generate the kurtogram of the filtered signal *y*(*n*). The 1/2-binary and 1/3-binary trees of filter-banks are obtained first and then implemented on the signal *y*(*n*). In consequence, a series of filtered signals is produced. Let $c_{i}^{k}$(*n*) be the sequence of the filtered signal issued from the *i*th filter at the *k*th level of the filter-bank tree, thus $c_{i}^{k}$(*n*) is the complex envelope of *y*(*n*) positioned on frequency *f_i_* and bandwidth (∆*f*)*_k_*. Then the kurtogram is constructed with the kurtosis values calculated by all the sequences $c_{i}^{k}$(*n*).
(3) Select the $c_{i}^{k}$(*n*) with the maximum kurtosis in the kurtogram and implement the envelope analysis on the sequence. As a result, the envelope spectrum with an optimal frequency band is obtained and it may demodulate the fault information buried in the original signal *x*(*n*).
(4) Detect the fault characteristic frequency in the envelope spectrum and diagnose the fault types.

## 4. Simulation Illustration

**Figure 2:**
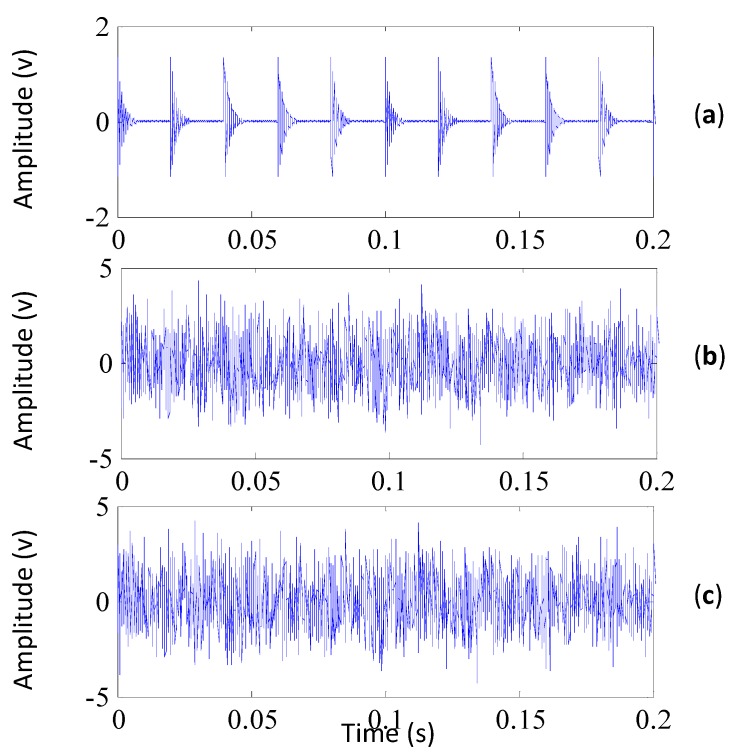
(**a**) The impulsive signal; (**b**) the noise signal; (**c**) the simulated signal (its kurtosis is 3.0).

In this section, a simulation of the early bearing fault diagnosis is conducted to illustrate the effectiveness of the proposed method. When faults occurred on bearings, the high-level periodic impulses are excited and decay exponentially [14], so the simulated signal consists of an impulsive signal and a noise signal. The impulsive signal is obtained by the following equation [15]:

(9) $$y(k)=\sum_{r}A_{r}\times \sin(2\text{π}f\times \left(k-r\times F/f_{\text{m}}\right)/F)\times e^{-\text{β}\times (k-r\times F/f_{\text{m}})/F}$$

where *A_r_* is the amplitude of the impulses and equals 1.5, *f_m_* is the fault characteristic frequency and equals 50 Hz, *F* is the sampling frequency which is set to 10 kHz, *f* is the resonant frequency equaling 2000 Hz and the decay parameter β is 500. A total of 20,000 samples are simulated for the impulsive signal. The noise signal refers to a white Gaussian noise with a mean of 0 and a variance of 1.5. We add the noise signal to the impulsive signal so as to generate the simulated signal. Figure 2 shows the impulsive signal, the noise signal and the simulated signal (to display these signals clearly, only 2000 samplings are shown). It can be seen from Figure 2c that the impulsive signal is totally overwhelmed by the noise signal, which is similar to the early fault signals of the bearings.

**Figure 3:**
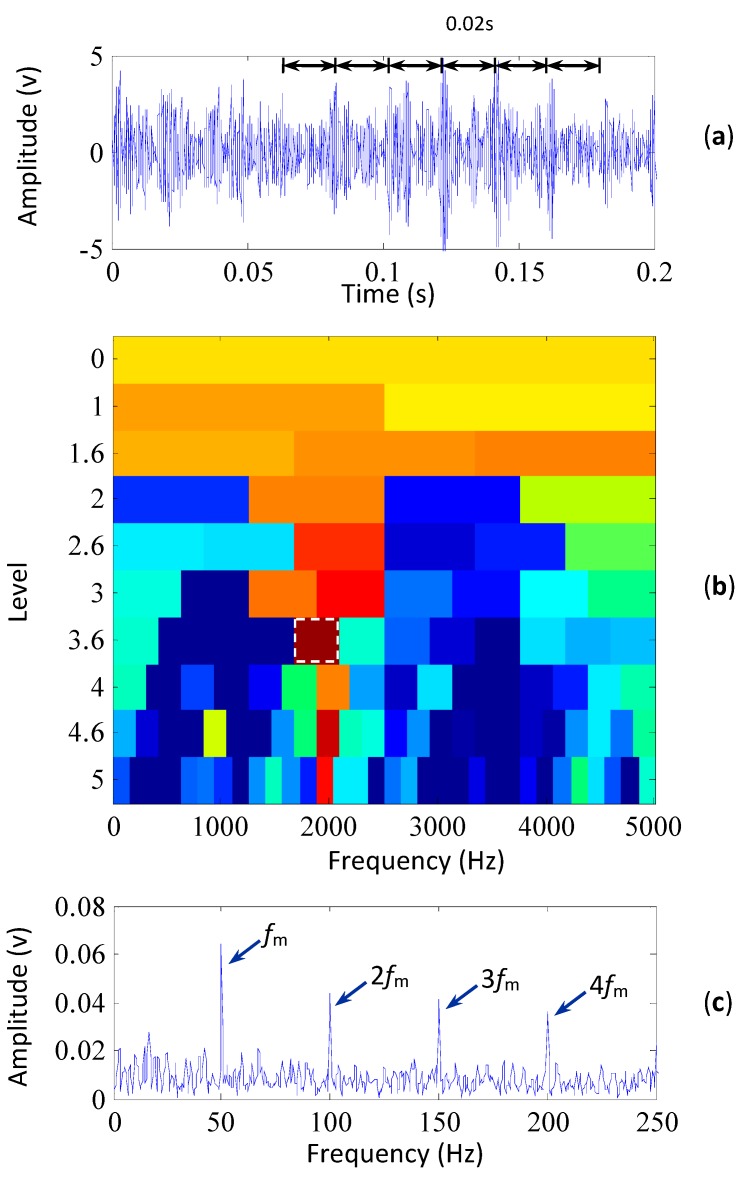
The results of the simulated signal based on the proposed method, (**a**) the signal preprocessed by MCKD (its kurtosis is 3.4); (**b**) the kurtogram of the preprocessed signal; (**c**) the envelope spectrum of the signal filtered by the selected frequency band.

The proposed method is employed to analyze the simulated signal. Figure 3a shows the signal preprocessed by MCKD. It can be seen that MCKD lifts the kurtosis value of the simulated signal from 3.0 to 3.4. Although the kurtosis value is not high, the MCKD helps clarify the impulses of the preprocessed signal. In Figure 2c, the impulsive signal is totally overwhelmed by the noise signal. After being preprocessed by MCKD, the impulses appear in the preprocessed signal. In Figure 3a, each interval of highlighted impulses is 0.02 s, corresponding to the simulated fault characteristic frequency. So the use of the MCKD can improve the performance of SK since it highlighted overwhelmed impulses to a level that reflects the fault. 

The kurtogram of the preprocessed signal using the proposed method is paved in Figure 3b and the frequency band with the highest kurtosis is indicated by the white rectangle. The center frequency and bandwidth of the selected frequency band are 1875 Hz and 416.7 Hz, respectively. It is noticed that the frequency band contains the resonant frequency of 2000 Hz. Then the simulated signal is filtered with the frequency band and its envelope spectrum is plotted in Figure 3c, where the fault characteristic frequency and its harmonics are evidently extracted. This illustrates that MCKD is able to improve the performance of SK in detecting the resonant frequency band so as to extract the fault characteristics from the signal overwhelmed by heavy noise. Therefore, the proposed method could not only detect the resonant frequency band, but also extract the fault characteristics from the signal overwhelmed by heavy noise.

**Figure 4:**
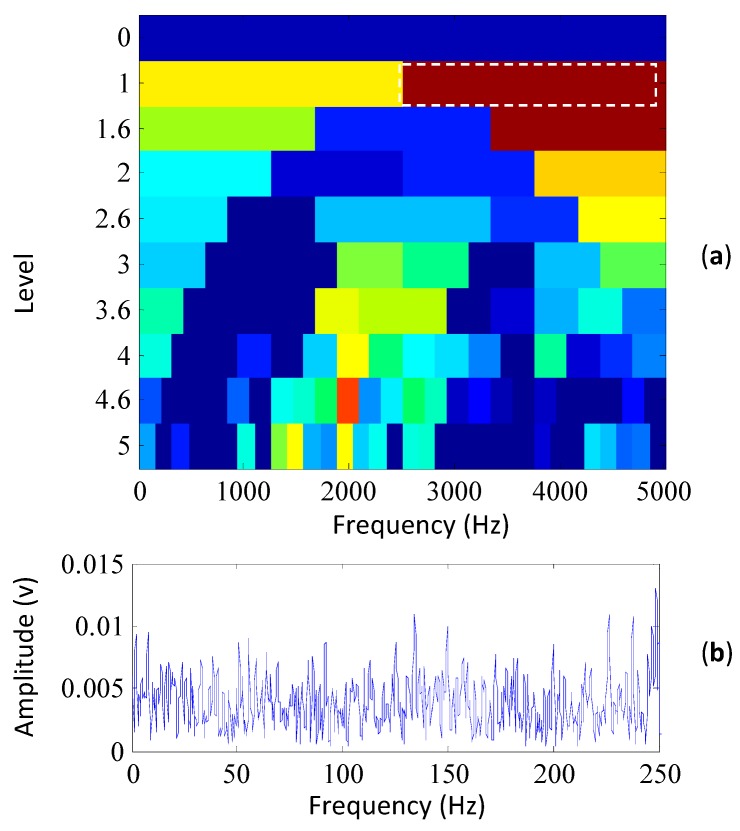
The results of the simulated signal based on the SK method, (**a**) its kurtogram; (**b**) the envelope spectrum of the signal filtered by the selected frequency band.

For comparison, the original SK method [12,13] is also applied to analyze the same simulated signal. Its kurtogram is shown in Figure 4a, in which the frequency band with the maximum kurtosis is indicated by the white rectangle. The frequency band has a center frequency of 3750 Hz and a bandwidth of 2500 Hz. The envelope spectrum of the frequency-band signal is shown in Figure 4b. From the figures, it can be seen that the SK method fails to detect the resonant frequency band and extract the fault characteristic frequency of the simulated signal. 

The comparison results demonstrate the superiority of the proposed method in extracting the fault characteristics from the simulated signals with a low signal-to-noise ratio. Therefore, the proposed method may effectively diagnose early faults of bearings in real cases.

## 5. Applications

### 5.1. Diagnosis of Wind Turbine Bearing Faults 

Due to its environmental friendliness, wind power has attracted much attention and become the fastest growing renewable energy source all over the world [20]. With a continuous increase in number and size of wind turbines, there is an urgent need to develop condition monitoring and fault diagnosis methods to avoid serious damage and reduce the operational cost. In this section, a real case is shown which uses the proposed method for diagnosing the bearing faults of a wind turbine. 

**Figure 5:**
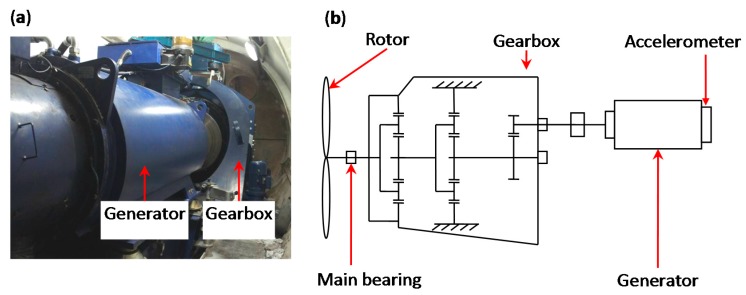
(**a**) A picture of the wind turbine; (**b**) structure sketch of the wind turbine and location of sensors.

The vibration signals were acquired from an operating wind turbine at a wind farm located in Zhangjiajie, China. The wind turbine is shown in Figure 5. It can be seen that with three blades supported by the main bearing, the rotor transmits the torque to the planetary gearbox through the low-speed shaft. Then the three-stage gearbox increases the rotational speed and transmits the torque to the high speed shaft, which drives the generator. When the maintenance workers checked the wind turbines during routine maintenance, they found that the vibration noise of the wind turbine was extremely heavy around its generator, but they failed to find the reasons for this abnormal phenomenon. Although it did not affect the operating status of the wind turbine, we considered that a fault occurred in the generator and began to monitor this wind turbine. We mounted accelerometers at the free-end of the generator. The accelerometer model used is the 356A12-type ICP accelerometer produced by PCB Piezotronics, Inc. (New York, NY, USA). The DT9837B module produced by Data Translation (Bietigheim-Bissingen, Germany) was employed for the data sampling of the voltage. A laptop with data acquisition software was used to collect the signals. The sampling frequency is 20,000 Hz. 

**Figure 6:**
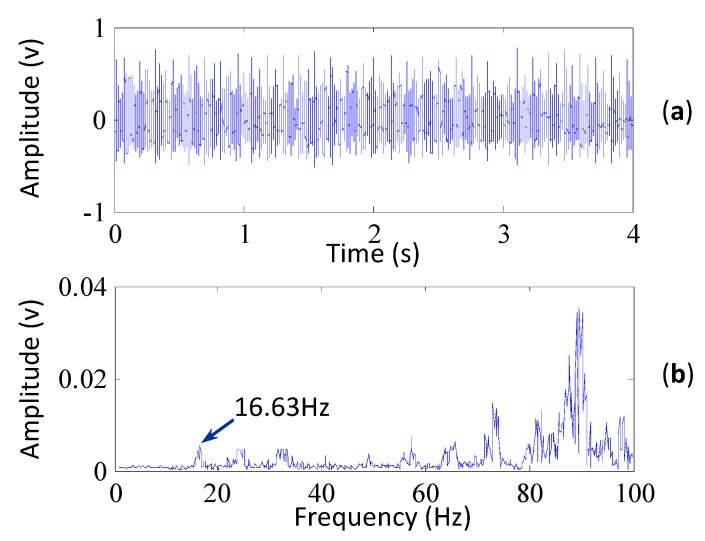
(**a**) The vibration signal of the wind turbine (its kurtosis is 3.14); (**b**) the frequency spectrum of the signal.

The defect frequencies of the bearings for the wind turbine bearing are displayed in Table 1. The vibration signal shown in Figure 6a is collected when the rotational speed of the high speed shaft is about 2.45 Hz. It is seen that weak impulses exist in the signal, which means there may be a fault in the generator. From the frequency spectrum in Figure 6b, it is found that there exists a large amplitude at 16.63 Hz, which corresponds to two times the fault characteristic frequency of the bearing outer race (the fault characteristic frequency of the outer race is *f_o_* = 2.45 × 3.38 = 8.281 Hz), but it is not evidence of the existence of faults, because the frequency component of 16.63 Hz is not a dominant component in the spectrum and the fault characteristic frequency of the outer race is not extracted.

**Table 1:** The defect frequencies of the wind turbine bearing (multiple of running speed in Hz).

Outer Race	Inner Race	Rolling Element	Rotational Speed of Shaft (Hz)
3.38	4.62	3.05	2.45

**Figure 7:**
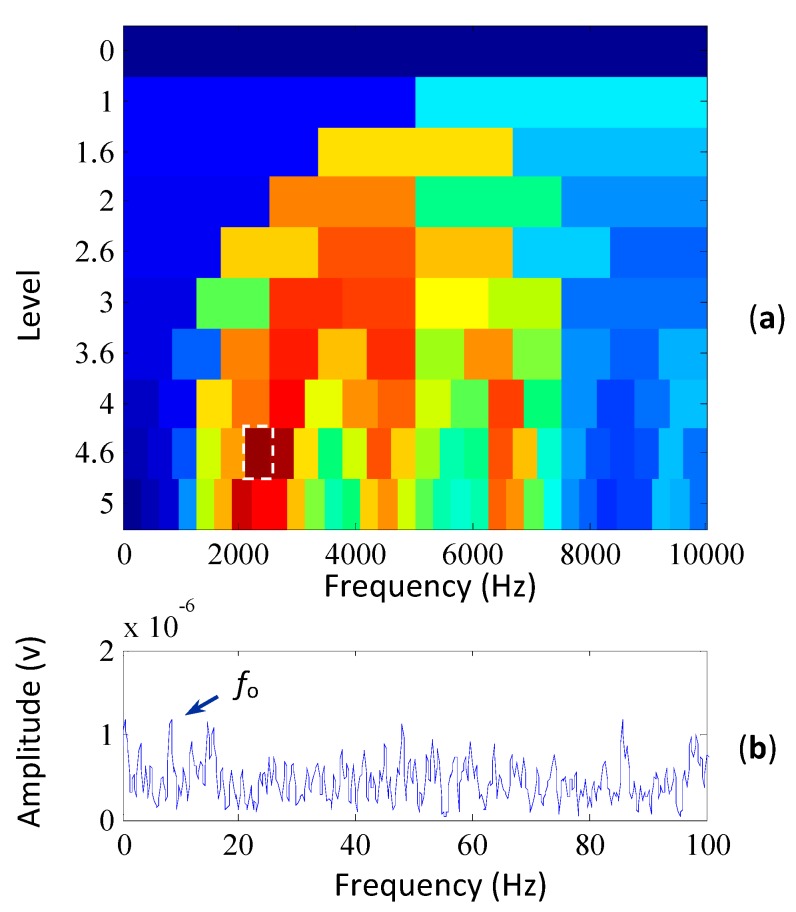
The results of the wind turbine signal based on the SK method. (**a**) its kurtogram; (**b**) the envelope spectrum of the signal filtered by the selected frequency band.

To confirm whether a fault occurred on the outer race, the SK method is applied to extract the weak fault characteristic frequency of the outer race. The corresponding kurtogram is plotted in Figure 7a and the frequency band with the maximum kurtosis is indicated by the white rectangle in the figure, which is selected as the optimal frequency band for filtering the signal. The filtered signal is further processed with the envelope analysis and its envelope spectrum is shown in Figure 7b. It can be seen from the envelope spectrum that the outer race fault frequency is extracted by the SK method. But the characteristic frequency is not evidence enough to confirm the existence of faults. 

**Figure 8:**
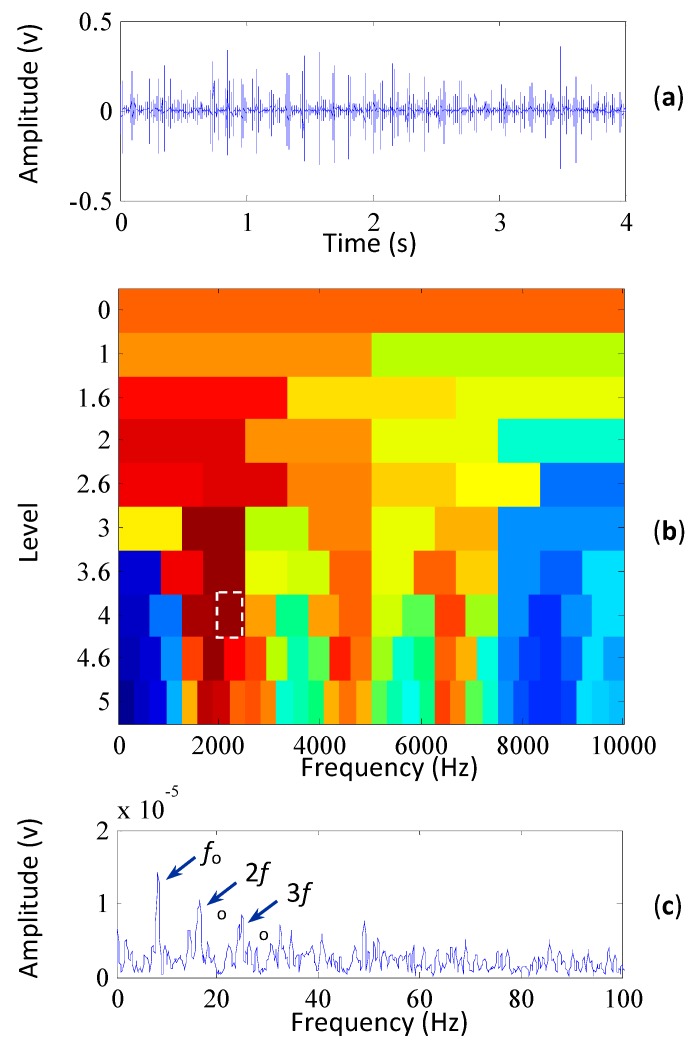
The results of the wind turbine signal based on the proposed method, (**a**) the signal preprocessed by MCKD (its kurtosis is 24.3); (**b**) its kurtogram; (**c**) the envelope spectrum of the signal filtered by the selected frequency band.

To extract the characteristic frequency evidently, the proposed method is employed to analyze the vibration signal. Its signal preprocessed by MCKD is shown in Figure 8a. It can be seen that by using MCKD the kurtosis of the signal dramatically increased from 3.14 to 24.3. The high kurtosis value of the preprocessed signal reflects the clarity of impulses. The kurtogram of the vibration signal based on the proposed method is shown in Figure 8b. The frequency band with the maximum kurtosis is indicated by the white rectangle, and Figure 8c shows the envelope spectrum of the corresponding frequency-band signal. It can be seen from the figure that the outer race fault frequency and its harmonics are extracted effectively, which illustrate that a fault occurred on the outer race of the bearing of the generator. Moreover, the signal-to-noise ratio in Figure 8c is much higher than that of Figure 7b, which demonstrates the superiority of the proposed method in extracting the fault characteristics of the bearings. 

### 5.2. Diagnosis of Rolling Mill Bearing Faults 

A rolling mill processes metal stock through one or more pairs of rollers to reduce the thickness and make the thickness uniform. As the key part of the rolling mill, the pinion stand has two output shafts which divide the power and deliver torque to the rolls. In metalworking, the bearings of the pinion stand often suffer from heavy load operating conditions, especially in the processes of occluding steel and tapping steel. Therefore, the bearings of the pinion stand are easily damaged, which may affect the rolling process. To guarantee the quality of the rolled steel, it is necessary to diagnose the early bearing faults of rolling mills.

**Figure 9:**
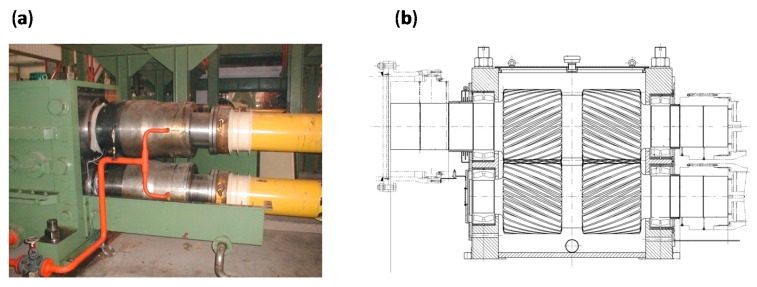
(**a**) A picture of the pinion stand; (**b**) structure sketch of its structure.

The case in this section uses the proposed method for diagnosing early bearing faults in the pinion stand of a hot strip rolling mill. A picture of the pinion stand and its structure are shown in Figure 9. The vibration signals were collected by an online data acquisition system, which was developed by the Iron and Steel Corporation (Taiyuan, China). The defect frequencies of the bearing are displayed in Table 2. It is seen that the shaft rotational frequency is *f*_r_ = 3.36 Hz, and the fault characteristic frequency of the inner race is *f*_i_ = 46.57 Hz.

**Table 2:** The defect frequencies of the rolling mill bearing (multiple of running speed in Hz).

Outer Race	Inner Race	Rolling Element	Rotational Speed of Shaft (Hz)
10.44	13.86	3.61	3.36

**Figure 10:**
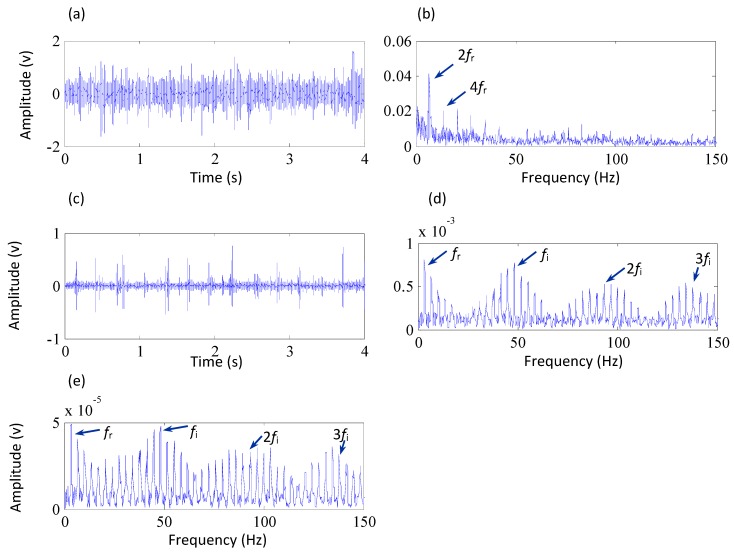
Analysis results of the signal collected from the pinion stand, (**a**) the original vibration signal (its kurtosis is 5.15); (**b**) the envelope spectrum of the original signal; (**c**) The preprocessed signal by MCKD (its kurtosis is 37.8); (**d**) the envelope spectrum based on the proposed method; (**e**) the envelope spectrum based on the SK method.

**Figure 11:**
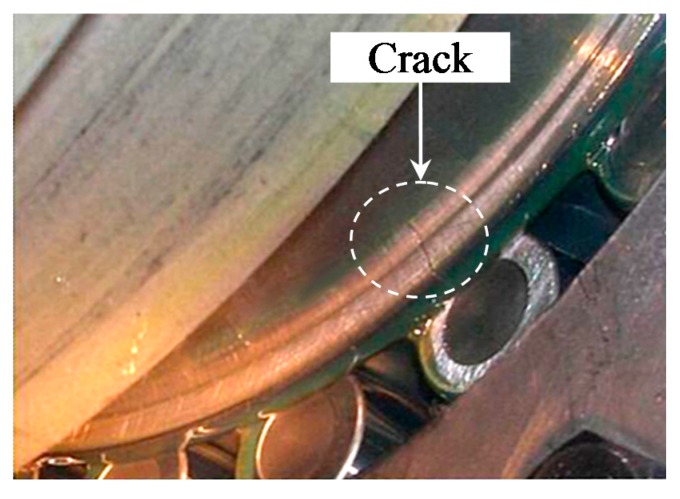
The crack fault occurred on the bearing inner race of the pinion stand.

Figure 10a shows the vibration signal collected from the pinion stand with a sampling frequency of 10 kHz. The figure reveals that the signal contains weak periodic components in the time domain, but the fault cannot be identified because of its weak characteristics. Consequently, the envelope analysis is used to demodulate the fault information from the signals and the result is shown in Figure 10b. In the figure, however, only two times the rotational frequency of the shaft *f*_r_ and its harmonics are clearly observed, instead of the fault characteristic frequency of the inner race *f*_i_. This illustrates that a fault may occur in the pinion stand but the fault cannot be determined by the results of the envelope analysis. Then the proposed method is applied to analyze the signal. As shown in Figure 10c, the kurtosis value of the signal preprocessed by MCKD increases to 37.8 compared to the value of 5.15 for the raw vibration signal, so the impulses of the signal are highlighted by using MCKD. The envelope spectrum based on the proposed method is shown in Figure 10d. It is observed that the fault characteristic frequency of the inner race and its harmonics are extracted by the proposed method and the intervals between the modulated sidebands are equal to the rotational frequency of the shaft. These results illustrate that a fault occurred on the inner race of the bearing and the proposed method is able to extract the weak fault characteristics effectively. To illustrate the effectiveness of the proposed method, a comparison is conducted with the SK method. Figure 10e shows the diagnosis result of the SK method. It can be noticed that the SK could also extract the inner race fault frequency and its harmonics, but other frequencies are also highlighted, which interferes the extraction of the fault characteristics. Thereby, it is verified that the proposed method performs better in identifying the early faults of the bearings. Additionally, Figure 11 shows that a crack fault has indeed occurred on the inner race of the bearing. 

## 6. Conclusions

This paper proposes an improved SK method based on MCKD for the early fault diagnosis of rolling element bearings. In the method, the main purpose of using MCKD is used to clarify the periodic impulse components of the bearing signals. It works well by selecting a FIR to maximize the correlated kurtosis of signals. SK could select a sensitive frequency band with a great quantity of impulses and demodulate the fault information from the frequency band. By analyzing the simulated and industrial monitoring signals, it is indicated that the proposed method could effectively suppress the heavy background noise and extract the weak fault characteristics from the vibration signals. Based on the comparisons with the SK method, it is verified that the proposed method has obvious advantages in the early fault diagnosis of the bearings.
